# The effect of the COVID-19 pandemic on pediatric emergency department utilization in three regions in Switzerland

**DOI:** 10.1186/s12245-024-00640-2

**Published:** 2024-05-16

**Authors:** Michael von Rhein, Aziz Chaouch, Vivian Oros, Sergio Manzano, Gianluca Gualco, Marc Sidler, Ursula Laasner, Michelle Dey, Julia Dratva, Michelle Seiler, on behalf of the PedCov consortium, Szilvia Altwicker-Hámori, Thomas Volken, Frank Wieber

**Affiliations:** 1 Child Development Center, University Children`s Hospital Zurich, University of Zurich, Zurich, Switzerland; 2 Department of Epidemiology and Health Systems, Center for Primary Care and Public Health (Unisanté), University of Lausanne, Lausanne, Switzerland; 3 University Children`S Hospital Zurich, University of Zurich, Zurich, Switzerland; 4 Pediatric Emergency Department, Geneva University Hospitals, University of Geneva, Geneva, Switzerland; 5 Pediatric Emergency Departement, Clinics of Pediatrics, Institute of Pediatrics of Southern Switzerland, EOC, Bellinzona, Switzerland; 6 Private Practice, Binningen, Switzerland; 7 Private Practice, Winterthur, Switzerland; 8 School of Health Science, ZHAW Zurich University of Applied Sciences, Winterthur, Switzerland; 9 Medical Faculty, University of Basel, Basel, Switzerland; 10 Pediatric Emergency Department, University Children`S Hospital Zurich, University of Zurich, Zurich, Switzerland

**Keywords:** Children, COVID-19, Emergency department, Pandemic, Utilization, Variation

## Abstract

**Purpose:**

The COVID-19 pandemic was associated with a decrease in emergency department (ED) visits. However, contradictory, and sparse data regarding children could not yet answer the question, how pediatric ED utilization evolved throughout the pandemic. Our objectives were to investigate the impact of the pandemic in three language regions of Switzerland by analyzing trends over time, describe regional differences, and address implications for future healthcare.

**Methods:**

We conducted a retrospective, longitudinal cohort study at three Swiss tertiary pediatric EDs (March 1st, 2018—February 28th, 2022), analyzing the numbers of ED visits (including patients` age, triage categories, and urgent vs. non-urgent cases). The impact of COVID-19 related non-pharmaceutical interventions (NPIs) on pediatric ED utilization was assessed by interrupted time series (ITS) modelling.

**Results:**

Based on 304′438 ED visits, we found a drop of nearly 50% at the onset of NPIs, followed by a gradual recovery. This primarily affected children 0–4 years, and both non-urgent and urgent cases. However, the decline in urgent visits appeared to be more pronounced in two centers compared to a third, where also hospitalization rates did not decrease significantly during the pandemic. A subgroup analysis showed a significant decrease in respiratory and gastrointestinal diseases, and an increase in the proportion of trauma patients during the pandemic.

**Conclusions:**

The COVID-19 pandemic had substantial effects on number and reasons for pediatric ED visits, particularly among children 0–4 years. Despite equal regulatory conditions, the utilization dynamics varied markedly between the three regions, highlighting the multifactorial modification of pediatric ED utilization during the pandemic. Furthermore, future policy decisions should take regional differences into account.

## What is known

While a substantial decrease in utilization of emergency department (ED) visits by adults has been described during the COVID-19 pandemic, There were contradictory reports regarding children.

## What is new

Based on more than 300′000 pediatric ED visits in three regions of Switzerland, we found a drop of nearly 50%, particularly in 0–4-year-old children, for both non-urgent and urgent medical conditions. Despite equal regulatory conditions, the utilization dynamics varied markedly between regions. Future policy decisions should take regional differences into account.

## Introduction

The first cases of Coronavirus disease 2019 (COVID-19) in Europe were recorded at the end of January 2020, with a subsequent rapid spread all over the continent and a significant rise in morbidity and mortality [1]. COVID-19 was declared a pandemic on March 11th 2020 by the World Health Organization (WHO), with 114 countries reporting more than 118,000 cases and 4,291 deaths due to the disease [2]. To combat the pandemic, public health measures, including social distancing, and hygiene measures were implemented in many countries. The strongest response to the pandemic were temporary nationwide non-pharmaceutical interventions (NPIs) imposed by the government with stay-at-home orders, school closures, and the shutdown of public life – in the public referred to as “lockdown”.

International evidence indicates that the COVID-19 pandemic caused significant changes in the utilization of health services [3–5]. Until 2020, the numbers of patient visits in pediatric emergency departments (EDs) had steadily increased over the last decades, culminating in severe overcrowding [6–9]. During the nationwide NPIs, pediatric ED visits remarkably dropped in numerous countries, with decreases ranging from 57% in Canada to 88% in Italy. This was mostly attributed to the stay-at-home policies and fear of catching or transmitting COVID-19 [10–13]. However, the reduction was greater in low-acuity triage scores, such as respiratory infections, injuries, and asthma exacerbations compared to high-acuity triage scores [5, 14]. An increase in high priority levels and the rate of hospitalizations at EDs indicated, that patients in urgent need of medical care still came to the ED despite the NPIs. Furthermore, some reports suggested, that delaying pediatric consultations to some extent lead to worsening of the patients` status before consulting the ED [11–15]. However, available publications on ED utilization are not all consistent, and vary in the observed effect sizes. For instance, in France it was shown that the number of visits for non-communicable infectious diseases was not different during a lockdown [16]. In most available publications, pre-pandemic data is compared with data from the time of nationwide NPIs [3–5, 11–17], reporting cross-sectional, single-center data over a short period of time. So far, only few studies used longitudinal analytical approaches to account for trends that already emerged in the pre-pandemic period, or trends across the pandemic phase [18–20]. However, most existing longitudinal research did not yet analyze data beyond the initial pandemic year (i.e., did not include data on utilization after lifting containment measures). Particularly studies from Switzerland are missing that compared objective and representative data on pre-pandemic and pandemic utilization of pediatric EDs.

Switzerland is nestled between Italy to the south and France to the west, both nations that were severely hit at the beginning of the pandemic [21, 22]. Accordingly, regional differences in mortalities within Switzerland were noted, with regions closest to Italy and France recording three times more COVID-19 deaths than the German speaking part of Switzerland by the end of the nationwide NPIs [23–26]. To prevent an uncontrolled,exponential spread of the epidemic and subsequent overburdening the healthcare system, the Swiss government introduced several public health measures to reduce transmission of COVID-19. On March 13, 2020, all schools were closed and elective and nonemergent medical care was restricted for all health care professions and levels of care, including pediatric care. A few days later (March 16), the most severe NPIs were enacted nationwide, corresponding a COVID-19 stringency index of 73 of 100 according to the *Oxford Covid-19 Government Response Tracker *[27]. Schools were reopened on May 11, the restriction on elective and nonemergent medical care lasted until May 27, 2020 (corresponding to a COVID-19 stringency index of 58, further dropping to 35 in June). Last restrictions were lifted completely in April 2022. These political decisions applied to all parts of the country.

The aim of this study was to longitudinally investigate the utilization of pediatric ED institutions during and over the COVID-19 pandemic and the installed containment measures in different regions of Switzerland, and address implications for future healthcare.

## Material and methods

This retrospective, longitudinal observational study was conducted at three tertiary pediatric EDs in Switzerland, representing the main language regions, from March 1, 2018 to February 28, 2022. Participating EDs were: one in the northern, German speaking part (Zurich, University Children’s Hospital), one in the western, French speaking part (Geneva, University Hospital), and one in the southern, Italian speaking part of Switzerland (Ticino, Pediatric Institute of Italian part of Switzerland).

Each patient visiting a pediatric ED, age 0 – 18 years old, at the participating centers was registered and an electronic medical file was created. The study utilized fully anonymized patient data from these files, including age, sex, day of ED registration, triage category, and type of treatment, as well as diagnoses in a subset of patients. The dataset obtained the following variables:

*Patient age*: the patient’s age was available in years, and then grouped into three categories: 0–4 years old, 5–12 years old, and 13–18 years old.

*Triage categories:* Triage categories are utilized to prioritize patient care according to their clinical urgency [28, 29]. The Australasian Triage Scale (ATS) was used in Zurich and Ticino, with five categories defined as follows: 1 = Immediately life-threatening, 2 = Imminently life-threatening (doctor contact within 10 min), 3 = Potentially life-threatening (doctor contact within 30 min), 4 = Potentially serious (doctor contact within 60 min), 5 = Less urgent (doctor contact within 120 min) [28]. The Canadian Triage and Acuity Scale (CTAS) was used in Geneva, consisting of the same five categories as the ATS, but with a difference in the time until doctor contact for triage scale 2, which was 15 min [29]. Both scales rate acuteness and severity in a very similar way [30]. For analysis purposes, triage scores 1–3, irrespective of triage scale, were considered as an urgent medical condition, while scores 4–5 were considered non-urgent.

*Type of treatment:* the type of treatment was categorized as outpatient or inpatient (i.e., hospital admission).

*Diagnoses:* Non-coded Diagnoses in 2019 and 2020 (only available in the German speaking part of Switzerland) were manually categorized into main categories: respiratory disease (e.g., infections, asthma), gastrointestinal disease (e.g., gastroenteritis, constipation), trauma (e.g., fracture, laceration, burn), skin disorders (e.g., rash, atopic dermatitis), infectious diseases others (e.g., soft tissue infections, osteoarticular infections, sepsis), nephrological/genital (e.g., infections, torsion of a testicle, glomerulonephritis), neurological (e.g., headache, syncope, seizure), mental health issues, foreign bodies, musculoskeletal/rheumatologic (non-traumatic disorders), cardiovascular (e.g., palpitation, heart failure), healthy, and others (e.g., hematological, allergic, postsurgical complications). Grouped diagnoses from March and April 2019 were compared to respective months in 2020.

### Statistical analyses

Interrupted time series (ITS) modelling was used to assess the COVID-19 related impact and compare pre-pandemic and pandemic health service consultations. The weekly number of ED visits in each cantonal hospital was calculated using the ISO 8601 standard to define weeks in the year. The first and last weeks of data (ISO week 9 in 2018 and 2022) were excluded as they had only partial counts, and week 53 of 2020 was also removed to ensure 52 weeks per year. The changepoint was set to week 12 of 2020 in all cantons, corresponding to the onset of the first nationwide NPIs on March 16th 2020. The pre-pandemic period was defined as the period preceding the changepoint, while the pandemic period was defined as the period following the changepoint. A transition period of ± 3 weeks around the changepoint (from week 9 to week 15 of 2020) was assumed, and data from this period were not used to fit the model.

This transition period was adopted to account for possible differences regarding the exact timepoint the changes in the number of ED visits would occur in the three cantons.

The ITS model was constructed using two negative binomial regressions (i.e., Poisson regression allowing for overdispersion), each fitted separately to each period. The expected counts were modelled on the logarithmic scale. Each regression included an intercept term and a linear trend (on log scale) for the time (in weeks) since the start of the observation period (i.e., week 10 of 2018), with time = 0 referring to the changepoint in week 12 of 2020. Within each period, seasonality was modelled using Fourier series with two harmonics. Additionally, residual autocorrelation was modelled using an autoregressive moving average (ARMA) process while assuming independence between data from the two periods. A suitable ARMA structure was selected by minimizing the corrected Akaike Information Criterion [31].

Interrupted time series (ITS) modelling was used to assess the COVID-19 related impact and compare pre-pandemic and pandemic health service consultations. The effects of interest (adjusted for seasonality) in the ITS model are as follows:

#### Time

Quantifies the pre-pandemic trend. The corresponding coefficient estimates the ratio of the expected number of visits (adjusted for seasonality) for two weeks separated by one year during the pre-pandemic period. A value of 1 indicates a stable condition (no change over time). A value above 1 suggests that the expected number of visits increased during the pre-pandemic period, while a value below 1 suggests that this number decreased.

#### Pandemic

Quantifies the magnitude of the drop in the number of visits at the onset of nationwide NPIs. The corresponding coefficient estimates the ratio in the expected number of visits (adjusted for seasonality) on week 12 of 2020 according to the pandemic and pre-pandemic models. A value of 1 indicates a stable condition (no change). A value above 1 suggests that the expected number of visits increased at the onset of nationwide NPIs, while a value below 1 suggests that this number decreased. Note that the percentage drop in the number of visits is quantified by one minus the pandemic coefficient.

#### Time x pandemic

Quantifies the difference between the pandemic and pre-pandemic trends (i.e., the interaction term in a regular ITS model). The corresponding coefficient estimates the ratio between the pandemic and pre-pandemic trends. A value of 1 indicates a stable condition (no change in trend between the pre-pandemic and pandemic periods). A value above 1 suggests that the pandemic trend increased compared to the pre-pandemic trend, while a value below 1 suggests that the pandemic trend decreased compared to the pre-pandemic trend.

Statistical analyses were conducted using R version 4.2.2 [32], and the gcmr package [33] was used to fit negative binomial regressions with autocorrelated errors. Categorical data were summarized by frequencies and percentages.

## Results

Over the study period from March 2018 to February 2022, a total of 304,438 ED visits were recorded (Zurich: 160,318, Geneva: 110,735, Ticino: and 33,385). The median age of patients visiting the EDs was 4 years (inter-quartile range: 1–9 years), with 54.7% of the children up to four years old, 35.1% aged 5–12 years, and 10.2% aged 13–18 years. Table 1 provides demographic information for the three EDs.

**Table 1:** Demographics

	Overall	Zurich	Geneva	Ticino
Number visits in ED	304′438	160′318	110′735	33′385
Age				
0–4 years (%)	166′578 (54.7)	91′976 (57.4)	60′823 (54.9)	13′779 (41.3)
5–12 years (%)	106′773 (35.1)	54′676 (34.1)	37′316 (33.7)	14′781 (44.3)
13–18 years (%)	31′087 (10.2)	13′666 (8.5)	12′596 (11.4)	4′825 (14.5)
Females (%)	136′974 (45.0)	72′336 (45.1)	50′005 (45.2)	14′633 (43.8)
Triage category urgent (%)	117′588 (38.6)	52′083 (32.5)	55′588 (50.2)	9′917 (29.7)
Hospitalizations (%)	30′757 (10.1)	18′690 (11.7)	9′350 (8.4)	2′717 (8.1)

*ED* emergency department

### Pre-pandemic trends

Regarding weekly ED visits and trends, ITS modeling revealed that after accounting for seasonality, the expected weekly number of visits in EDs during the pre-pandemic period remained relatively stable in Zurich, while numbers increased by approximately 6% every year in Geneva and Ticino. Taken together, these increases were not statistically significant. However, when focusing on urgent visits, significant pre-pandemic trends were observed in Geneva (+ 10.1% per year, 95% CI [1.2; 19.8]) and Ticino (+ 38.7% per year, 95% CI [22.3; 57.3]), compared to a comparably stable situation in Zurich (+ 4.8% per year, 95% CI [-3.5; 13.9]). In Geneva and Ticino, positive pre-pandemic trends were also observed in the number of hospitalizations (+ 5.1% per year in Geneva, 95% CI [0.5; 9.9], and + 21.6% per year in Ticino, 95% CI [8.5; 36.3]), compared to Zurich (-0.1% per year, 95% CI [-4.8; 4.8]).

### Effects of the pandemic

**Fig. 1:**
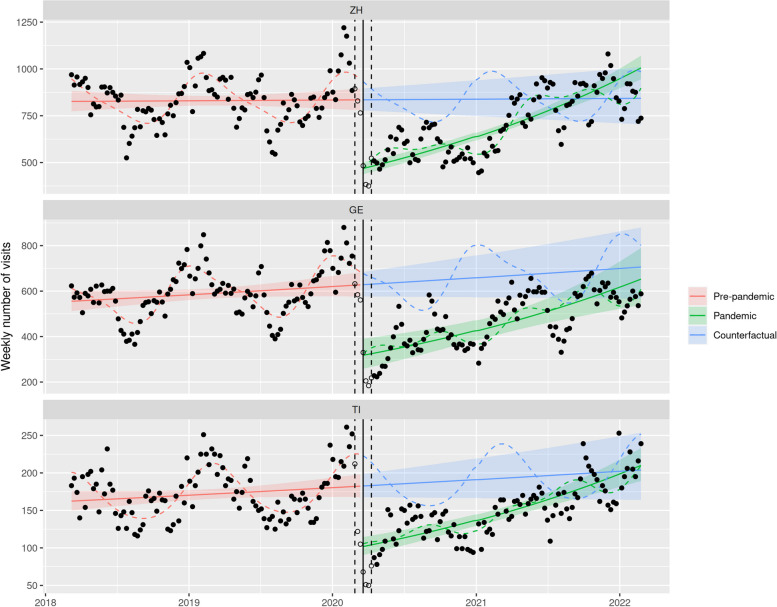
ITS models of total ED visits in the different language regions of Switzerland. *Legend:* Red: ITS model of pre-pandemic ED visits (March 1st to February 24th 2018), green: ITS model of pandemic ED visits (April 6th to February 28th 2022), blue: counterfactual extrapolation of the further course of ED visits based on the ITS model of pre-pandemic data, solid line: onset of the first phase of nationwide NPIs on March 16th 2020, dashed line: transition period of ± 3 weeks around March 16.^th^. ZH: Zurich (German speaking part), GE: Geneva (French speaking part), TI: Ticino (Italian speaking part)

**Fig. 2:**
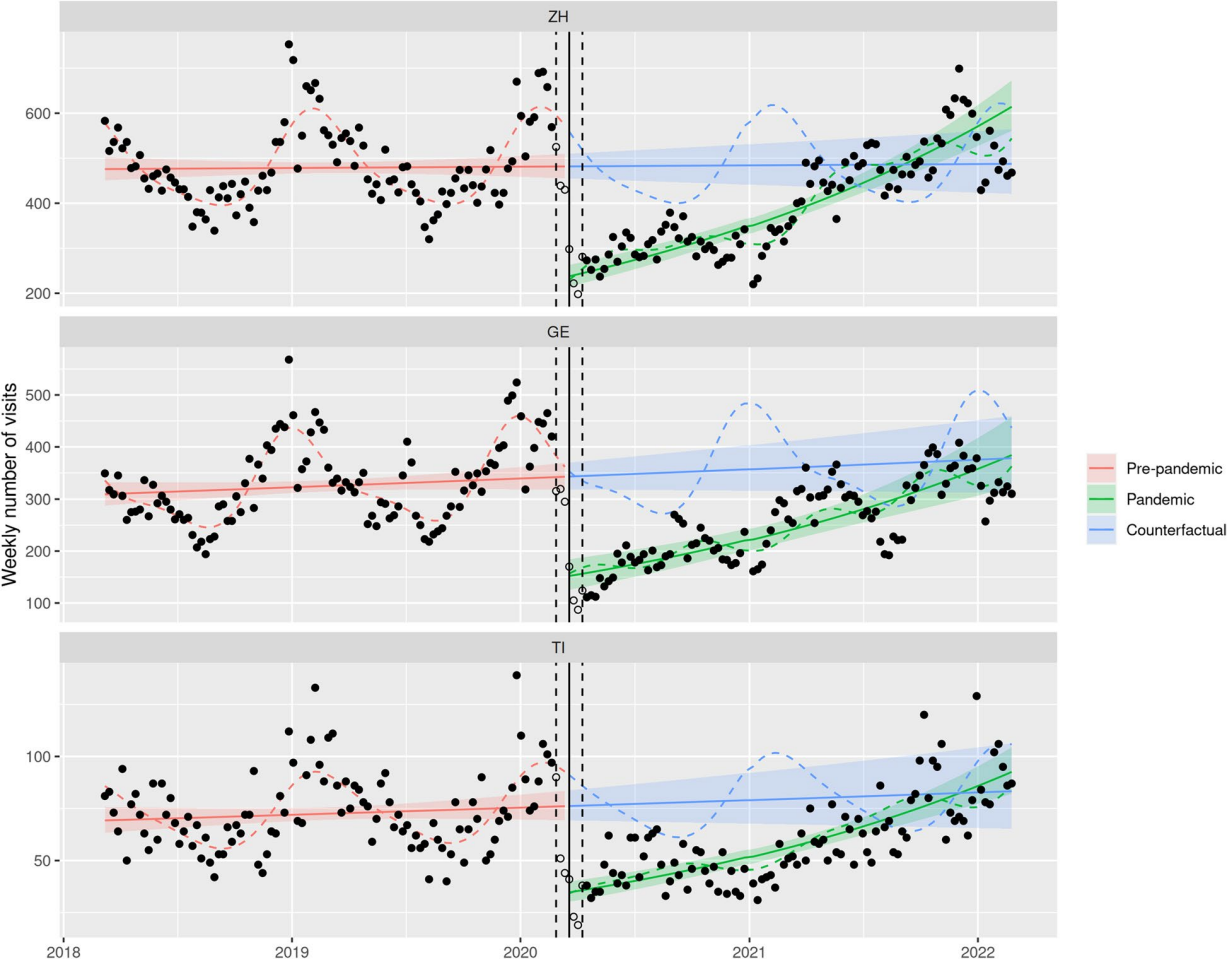
ITS models of ED visits of children 0–4 years old per language region. *Legend:* Red: ITS model of pre-pandemic ED visits (March 1st to February 24th 2018), green: ITS model of pandemic ED visits (April 6th to February 28th 2022), blue: counterfactual extrapolation of the further course of ED visits based on the ITS model of pre-pandemic data, solid line: onset of the first phase of nationwide NPIs on March 16th 2020, dashed line: transition period of ± 3 weeks around March 16.^th^. ZH: Zurich (German speaking part), GE: Geneva (French speaking part), TI: Ticino (Italian speaking part)

The ITS models revealed a decrease of nearly 50% in the number of ED visits at the onset of nationwide NPIs (-43.9% in Zurich, 95% CI [-38.1; -49.1], -49.4% in Geneva, 95% CI [-36.7; -59.6], -44.2% in Ticino, 95% CI [-35.5; -51.7]). This drop began even slightly before the nationwide NPIs officially started (March 13th 2020), and was followed by a gradual recovery until the second half of 2021 when the number of ED visits reached pre-pandemic levels (Fig. 1 and Table 2). These trends were consistent across all three regions and mainly affected the youngest age group, with a drop exceeding 50% (Fig. 2 and Table 3). In the age groups of 5–12 years old, a smaller drop was observed (-35.5% in Zurich, 95% CI [-30.7; -40.0],—43.0% in Geneva, 95% CI [-28.7; -54.4], -38.0% in Ticino, 95% CI [-29.5; -45.6]). In the age group of 13–18 years old, the reduction was even smaller, but still significant (-22.9% in Zurich, 95% CI [-7.6; -35.6], -34.3% in Geneva, 95% CI [-12.4; -50.7], -26.9% in Ticino, 95% CI [-11.5; -39.7]). However, the time courses of these decreases were not entirely synchronous: for instance, the observed number of visits in the ED in Ticino already dropped drastically in the two weeks preceding the onset of nationwide NPIs.

**Table 2:** Model estimates for the weekly total number of visits in emergency departments in Zurich (ZH), Geneva (GE) and Ticino (TI)

Canton	Time	Pandemic	Time x Pandemic
ZH	1.005 [0.947; 1.067]	0.561*** [0.509; 0.619]	1.480*** [1.359; 1.612]
GE	1.062 [0.988; 1.143]	0.506*** [0.404; 0.633]	1.369** [1.125; 1.665]
TI	1.059 [0.985; 1.139]	0.558*** [0.483; 0.645]	1.376*** [1.214; 1.560]

Interrupted time-series negative binomial regression, adjusted for seasonality and autocorrelation. Coefficients refer to the ratio of weekly number of visits (after one year for trends). 95% confidence interval in brackets

^*^p < 0.05

^**^p < 0.01

^***^p < 0.001

**Table 3:** Model estimates for emergency department number of visits (subgroups) in Zurich (ZH), Geneva (GE), and Ticino (TI)

Canton	Time	Pandemic	Time x Pandemic
Models including children 0–4 years old only			
ZH	1.006 [0.959; 1.056]	0.493*** [0.439; 0.554]	1.628*** [1.472; 1.802]
GE	1.052 [0.987; 1.122]	0.444*** [0.361; 0.545]	1.539*** [1.284; 1.844]
TI	1.048 [0.966; 1.136]	0.456*** [0.385; 0.539]	1.590*** [1.376; 1.837]
Models including urgent cases only			
ZH	1.048 [0.965; 1.139]	0.573*** [0.498; 0.659]	1.444*** [1.279; 1.631]
GE	1.101* [1.012; 1.198]	0.487*** [0.375; 0.632]	1.476*** [1.176; 1.854]
TI	1.387*** [1.223; 1.573]	0.676*** [0.570; 0.802]	1.027 [0.885; 1.193]
Models including hospitalized patients only			
ZH	0.999 [0.952; 1.048]	0.749*** [0.693; 0.808]	1.186*** [1.111; 1.267]
GE	1.051* [1.005; 1.099]	0.593*** [0.481; 0.732]	1.396*** [1.166; 1.670]
TI	1.216*** [1.085; 1.363]	0.989 [0.822; 1.190]	0.790** [0.671; 0.932]

Interrupted time-series negative binomial regression, adjusted for seasonality and autocorrelation. Coefficients refer to the ratio of weekly number of visits (after one year for trends). 95% confidence interval in brackets

^*^p < 0.05

^**^p < 0.01

^***^p < 0.001

**Fig. 3:**
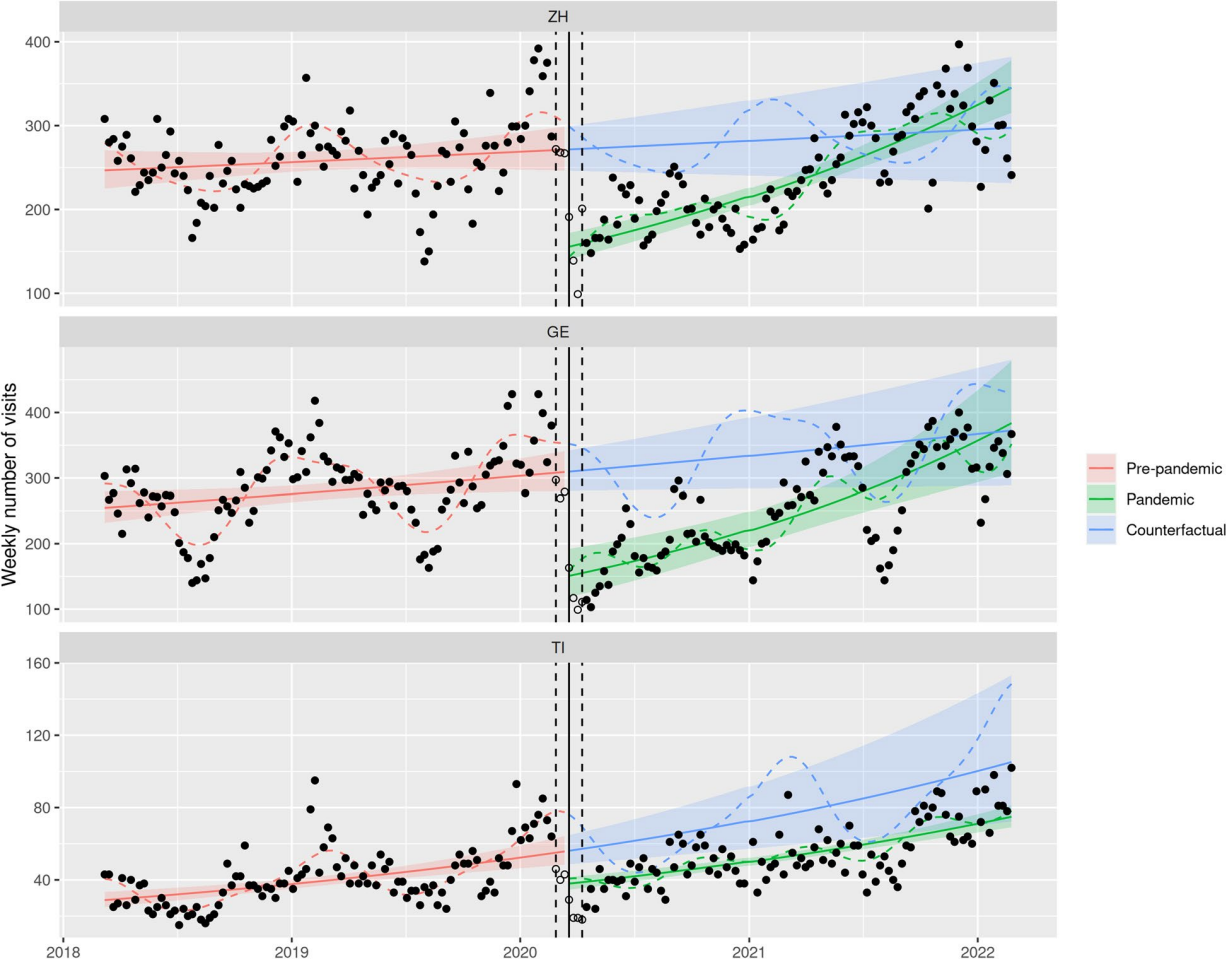
ITS models of ED visits of urgent cases (triage scores 1–3) per language region. *Legend:* Red: ITS model of pre-pandemic ED visits (March 1st to February 24th 2018), green: ITS model of pandemic ED visits (April 6th to February 28th 2022), blue: counterfactual extrapolation of the further course of ED visits based on the ITS model of pre-pandemic data, solid line: onset of the first phase of nationwide NPIs on March 16th 2020, dashed line: transition period of ± 3 weeks around March 16.^th^. ZH: Zurich (German speaking part), GE: Geneva (French speaking part), TI: Ticino (Italian speaking part)

**Fig. 4:**
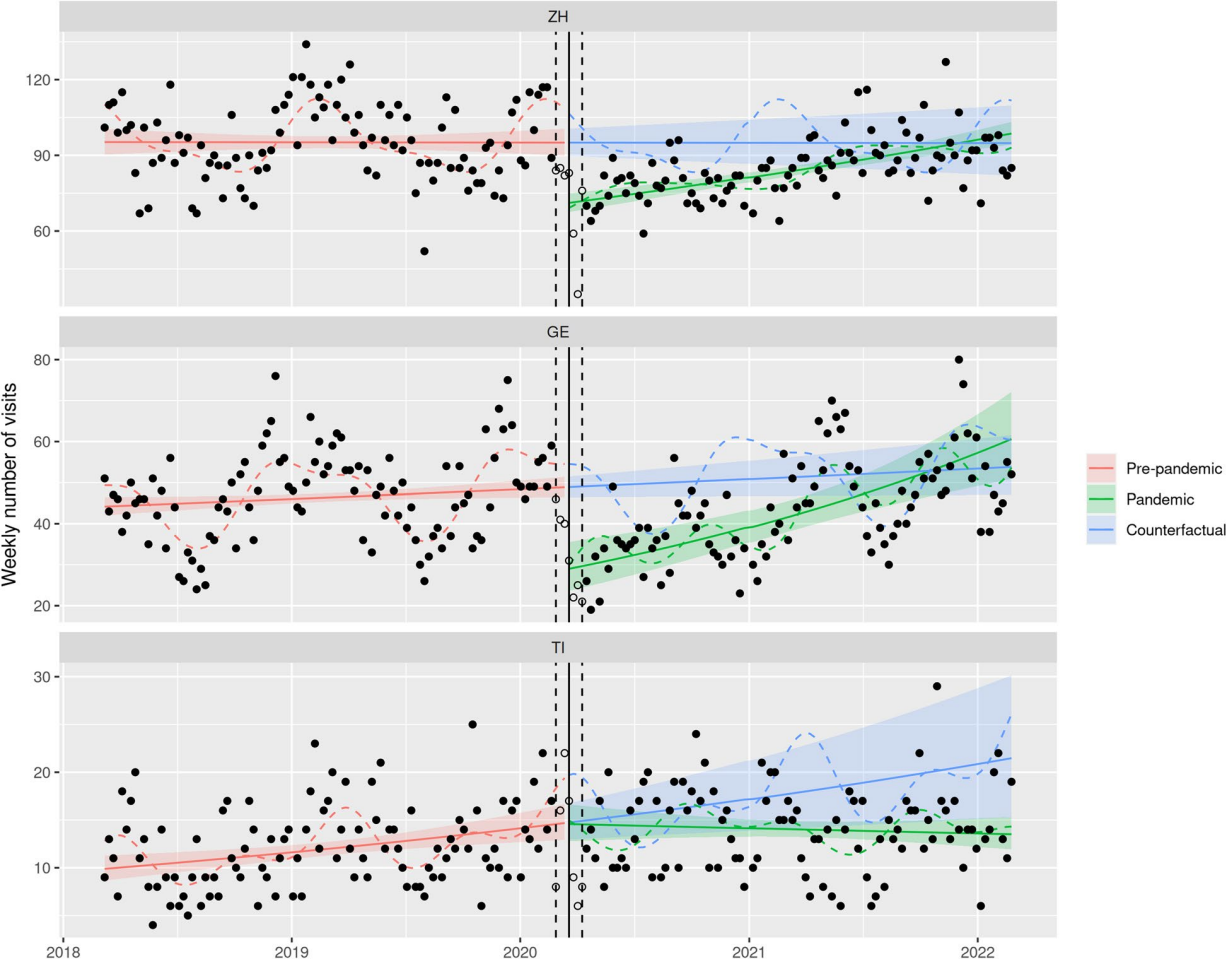
ITS models of ED leading to hospitalization per language region. Legend: Red: ITS model of pre-pandemic ED visits (March 1st to February 24th 2018), green: ITS model of pandemic ED visits (April 6th to February 28th 2022), blue: counterfactual extrapolation of the further course of ED visits based on the ITS model of pre-pandemic data, solid line: onset of the first phase of nationwide NPIs on March 16th 2020, dashed line: transition period of ± 3 weeks around March 16th. ZH: Zurich (German speaking part), GE: Geneva (French speaking part), TI: Ticino (Italian speaking part)

Both urgent and non-urgent cases decreased at the onset of nationwide NPIs. Non-urgent cases significantly dropped by 45.0% in Zurich (95% CI [37.3; 51.8],]), 47.4% in Geneva (95% CI [36.4; 56.5]) and 49.8% in Ticino (95% CI [42.2; 56.4]). However, the drop in urgent cases was not as pronounced in Ticino (33.4%) compared to Zurich (42.7%) and Geneva (51.3%) as illustrated in Fig. 3 and Table 3. The number of patients with outpatient care significantly dropped in all regions (-46.4% in Zurich, 95% CI [-38.7; -53.0], -50.1% in Geneva, 95% CI [-37.5; -60.2], -47.6% in Ticino, 95% CI [-39.2; -54.8], data not shown), whereas hospitalizations only decreased at the onset of NPIs in Zurich and Geneva (-25.1% in Zurich, 95% CI [-19.2; -30.7], -40.7% in Geneva, 95% CI [-26.8; -51.9], -1.1% in Ticino, 95% CI [-19.0; 17.8], see Fig. 4).

A gradual catch-up in the number of visits was observed during the pandemic period following nationwide NPIs for most patient groups, but some notable exceptions were identified by the ITS models. First, in contrast to what happened in Geneva and Zurich, the increase in the number of urgent visits in Ticino was not different from that observed during the pre-pandemic period (Fig. 3). Secondly, hospitalizations in Ticino even gradually decreased after the NPIs were released (Fig. 4 and Table 3).

### Diagnoses

Grouped diagnoses were evaluated to determine changes in the prevalence of specific medial conditions during the months of March and April from 2019–2020 in the largest subgroup (German speaking part of Switzerland). A detailed analysis of diagnostic groups revealed a significant decrease in respiratory and gastrointestinal diseases in 2020 compared to 2019. As a proportion of visits, communicable diseases such as upper and lower airway infections and gastroenteritis were found to have dropped from 33.4% to 26.5% and from 17.7% to 12.0%, respectively between 2019 and 2020. In contrast, the proportion of visits for trauma during the same period significantly increased from 27.2% in 2019 to 36.3% in 2020 (*p* < 0.001), despite a drop of the total number of trauma related visits (Table 4). No changes were observed in the proportion of cardiovascular diseases and mental health problems.

**Table 4:** Number of pediatric ED visits (%) in March and April of 2019 and 2020 by diagnostic group

	**March/April 2019**	**March/April 2020**	***P*-value**
Total	7,707	4,776	
Diagnostic groups			
respiratory disease	2,572 (33.4)	1,264 (26.5)	< 0.001
gastrointestinal disease	1,365 (17.7)	574 (12.0)	< 0.001
trauma	2,096 (27.2)	1,734 (36.3)	< 0.001
skin disorders	309 (4.0)	174 (3.6)	0.326
nephrological/genital	252 (3.3)	220 (4.6)	< 0.001
musculoskeletal/rheumatologic	59 (0.8)	37 (0.8)	1.000
cardiovascular	44 (0.6)	31 (0.6)	0.667
neurological	220 (2.9)	158 (3.3)	0.166
mental	108 (1.4)	68 (1.4)	0.979
infectious diseases (others)	259 (3.4)	188 (3.9)	0.102
foreign bodies	97 (1.3)	82 (1.7)	0.044
others	211 (2.7)	178 (3.7)	0.002
healthy	115 (1.5)	68 (1.4)	0.816

## Discussion

The aim of this study was to investigate the utilization of pediatric ED institutions during the COVID-19 pandemic and the installed containment measures in different regions of Switzerland. At the onset of nationwide NPIs, a drop of nearly 50% in the number of ED visits was observed, followed by a gradual catch-up until the second half of 2021 when the number of ED visits reached pre-pandemic levels. This pattern mostly affected the youngest age group (0–4 years old) and was similar for patients with non-urgent and urgent medical conditions in all three regions. However, the decrease in urgent visits appeared more pronounced in Zurich and Geneva than in Ticino. Accordingly, hospitalization rates in the Italian speaking part of Switzerland did not decrease significantly during the pandemic, in contrast to the findings in the German and French speaking parts. In-depth analyses of diagnostic groups revealed a significant decrease in the proportion of viral infections (respiratory and gastrointestinal) in 2020 compared with 2019, whereas the proportion of trauma patients increased significantly.

### Dynamics of pediatric ED utilization

The COVID-19 related nationwide NPIs resulted in a nearly 50% decrease in ED visits in Swiss pediatric EDs, which is consistent with findings from international research reporting reductions ranging from 30 to 89% [11–15, 34, 35]. We observed that, pediatric ED visits began to drop even before the nationwide NPIs started (March 13th 2020), as the first positive COVID-19 cases in Switzerland were reported. The time needed until pediatric ED visits reached pre-pandemic levels lasted over a year (spring 2020 to summer 2021) in our study. These results are consistent with an analysis from Portugal, which reported a catch-up of ED visits after stopping NPIs, but did not reach pre-pandemic levels by July 2021 [36]. A US study even found that visits had not yet normalized until January 2022 [23]. The reasons for this may include parents’ fear of contracting COVID-19 in EDs [36–38], fewer infections due to containment measures such as wearing face masks at public gatherings [38], and longer lasting and more drastic COVID-19 measures due to a more severe course of the pandemic in the two neighboring countries.

The reduction in pediatric ED visits mostly affected the youngest age group, which usually accounts for the largest proportion of patients in pediatric EDs [39]. Similar results were found by Mataloni et al. [35], who reported a decrease of 50% in children up to the age of five years. The most common diagnoses in pediatric EDs are breathing difficulties, febrile illnesses, and gastroenterits [39], which mostly affect young children [40]. These diseases declined during and after the nationwide installation of NPIs [38, 41]. The lower rates of communicable diseases in this age group were probably caused by fewer contacts with other children and less parent- or siblings-to-child-transmission because of hygiene and containment measures. Whether the observed decrease in ED visits among this age group represents a genuine decline in disease is uncertain, as it is possible that care has shifted to alternative settings. Parents might have opted to avoid visiting EDs with their children too young to wear face masks, and instead chose to visit a pediatrician in private practice, use telemedicine, or just waited for the natural course of the disease without seeking professional aid at pediatric EDs.

### Regional variations of effects on different triage and age groups

Urgent and non-urgent cases decreased at the onset of nationwide NPIs in all regions. While the reduction rates for both urgent and non-urgent cases were comparable in Zurich in the French speaking part of Switzerland, the Italian speaking part experienced the most significant drop of non-urgent cases (-49.8%), whereas the decline in urgent cases was less pronounced (-32.4%). These findings are consistent with Italian studies, which have reported notable reductions in non-urgent cases but an increased rate of urgent cases and hospitalizations [4, 11, 12, 14]. Consequently, the rate of hospitalizations did not decrease at the beginning of the pandemic in the Italian speaking region, unlike the German and French speaking part of the country. This disparity may be attributed to the significantly higher incidence of COVID-19 positive cases in the Italian speaking part of Switzerland and bordering north of Italy compared to the other parts of the country during the initial phase of the pandemic. When comparing inpatient and outpatient care, the decrease was very similar for both modalities in Geneva (inpatients: -40.7%, outpatients: -50.1%), but it was most divergent in Ticino (inpatients -1.1%, outpatients -47.6%). Contrary to Zurich and Geneva, Ticino already experienced a pre-pandemic increase in the number of urgent visits which remained unchanged during the pandemic.

Despite official recommendations on pediatric health care, pediatric ED visits halved after the nationwide installation of NPIs in all three language regions, primarily impacting infants and toddlers. Interestingly, we observed clear differences between the three language regions, while equal regulations were in place. In contrast to the number of hospitalizations in the German and French speaking parts of Switzerland, those in the Italian speaking part of the country remained at the pre-pandemic level. Furthermore, the drop of ED visits happened earlier in the Italian speaking part than in the other regions. These regional differences illustrate that, despite equal regulations and rules, utilization behavior can vary considerably—presumably modified by various factors (e.g., differences in the regional severity of the pandemic, local incidences of diseases, as well as attitude, or habits of the respective populations). Therefore, approaches that recognize regional differences rather than global measures may be needed in order to appropriately react to large scale challenges.

### Shift of diagnoses over time

Among critically ill patients, seizures, bronchiolitis, and asthma account for the most common diagnoses in pediatric EDs [40]. Since detailed analyses of diagnoses were not possible for the Italian and French speaking part of Switzerland due to unavailable data, changes in the diagnoses of ED patients in Zurich were analyzed. We found that the proportions of respiratory and gastrointestinal diseases decreased, whereas trauma cases proportionally increased during the NPIs. International studies have also reported a significant decline in respiratory and gastrointestinal infections, primarily attributed to reduced opportunities for transmission due to school closures and social distancing measures [3–5, 11, 14, 17, 38]. Additionally, the reduction in air pollution resulting from decreased traffic contributed to a reduction of asthma exacerbations [42, 43]. Finally, besides a true decrease in incidence, the lower number of children presenting with viral infections might also in part be explained by their parents` hesitancy to visit an ED due to parental concern about COVID infection leading to later presentation of their sick children to a pediatrician, as described by Davis et al. [10]. Recent publications on trauma cases present inconsistent findings, with some reporting a decline and others reporting an increase in the rate of cases [38, 43]. We found only a minor reduction in absolute numbers of trauma patients, but a 10% increase in the proportion of trauma patients among all ED visits after the end of nationwide NPIs. This stable absolute number is what we would expect given that in Switzerland children were allowed to go outside and outdoor playgrounds were not closed, which might have increased the number of accidents. Furthermore, home accidents may have increased due to less supervision of young children as parents were occupied with working from home, while schools and child care were closed [43], even though the implementation of a stay-at-home policy resulted in fewer accidents due to cancelled sporting events [38]. However, the proportional increase mainly stems from the fact, that other diagnoses were less frequent.

### Strengths and limitations

This study has several limitations. Although we analyzed the largest pediatric EDs in each language region, the generalizability of the results to other pediatric hospitals in the respective language region as a whole may be limited as disparities of the populations served might exist. We found no increase of mental health problems, which might be due to the limited period of time we analyzed. In fact, the proportion of mental health issues might have increased later without being captured. Furthermore, our dataset does not cover the post-NPI period (NPIs were completely lifted in April 2022 in Switzerland), as we aimed to primarily focus on the changes during the initial phase of the pandemic and the nationwide start of NPIs in our report. Additionally, diagnoses were only available for Zurich and not Geneva and Ticino. Possible differences between diagnoses in these regions therefore would remain undetected. Also, the number of visits in Ticino are fairly low compared to those in Zurich and Geneva. This must be taken into account in order not to overinterpret observed differences. Nonetheless, the study’s strength lies in its large dataset and longitudinal observations spanning four years, which enabled us to assess the impact of the pandemic. Combining our data with health insurance data on pediatric outpatient health care use during the same time period (such analyses are planned by the PedCov consortium) will help to get further insights into utilization patterns during the pandemic.

The COVID-19 pandemic had enormous implications on healthcare, providing important lessons for future healthcare crises. Our study shows significant changes on pediatric ED utilization, but also regional differences, that call for tailored and dynamic management during future comparable challenges for the health care system. Real-time monitoring not only of positive SARS COV-2 cases, but also of other indicators (e.g., number of well-child visits or vaccinations) might have helped to identify trends in parents` health-related attitudes and behavior even at a regional level, and adapt policies or communicate with the public, accordingly. The three pediatric EDs in our study experienced an unprecedented reduction in visits, which can be partly attributed to the implementation of hygiene measures, and a stay-at-home policy. However, the variations between the participating centers and the fact that reaching pre-pandemic levels took more than a year after lifting restrictions in May 2020 highlight that also other factors like parents` attitudes or habits might play a role in modifying health care utilization. Therefore, health care policies should acknowledge regional differences when deciding on measures in reaction to similar future events. This is particularly true since the installed containment measures also posed risks to children, as delayed ED presentations could lead to serious consequences. New ways to counsel parents, or more successful communication strategies between the authorities and the public might help to better react to future challenges of the pediatric health care sector.

## Data Availability

The study results we report here have already been presented on, or submitted as an abstract to scientific conferences (annual meeting of the Swiss and German pediatric societies 2023), but have not been otherwise published yet. Individual participant data cannot be shared, but a data dictionary can be provided upon request to the corresponding author. The study protocol, and statistical analyses performed are described in the methods section of the manuscript.

## Abbreviations

ARMA: Autoregressive moving average
ATS: Australasian Triage Scale
CTAS: Canadian Triage and Acuity Scale
CI: Confidence Interval
ED: Emergency departments
NPIs: Non-pharmaceutical Interventions
WHO: World Health Organization
